# Reduction of the Oxidative Stress Status Using Steviol Glycosides in a Fish Model* (Cyprinus carpio)*

**DOI:** 10.1155/2017/2352594

**Published:** 2017-06-12

**Authors:** Livier Mireya Sánchez-Aceves, Octavio Dublán-García, Leticia-Xochitl López-Martínez, Karen Adriana Novoa-Luna, Hariz Islas-Flores, Marcela Galar-Martínez, Sandra García-Medina, María Dolores Hernández-Navarro, Leobardo Manuel Gómez-Oliván

**Affiliations:** ^1^Laboratorio de Toxicología Ambiental, Facultad de Química, Universidad Autónoma del Estado de México, Paseo Colón Intersección Paseo Tollocan s/n, Col. Residencial Colón, 50120 Toluca, MEX, Mexico; ^2^Centro de Investigación en Alimentación y Desarrollo, A. C. Unidad Culiacán, Carretera El Dorado Km 5.5, Col. Campo El Diez, 80110 Culiacán, SIN, Mexico; ^3^Laboratorio de Toxicología Acuática, Departamento de Farmacia, Escuela Nacional de Ciencias Biológicas, Instituto Politécnico Nacional, Unidad Profesional Adolfo López Mateos, Av. Wilfrido Massieu Esq. Cda. Miguel Stampa s/n, Delegación Gustavo A. Madero, 07738 Ciudad de México, Mexico

## Abstract

Steviol glycosides are sweetening compounds from the* Stevia rebaudiana* Bertoni plant. This product is considered safe for human consumption and was approved as a food additive by the Food and Drugs Administration (FDA) and European Food Safety Authority (EFSA). Its effects on the ecosystem have not been studied in depth; therefore, it is necessary to carry out ecotoxicological studies in organisms such as* Cyprinus carpio*. The present study aimed to evaluate the antioxidant activity by SGs on diverse tissues in* C. carpio* using oxidative stress (OS) biomarkers. To test the antioxidant activity, carps were exposed to four systems: (1) SGs free control, (2) CCl_4_ 0.5 mL/kg, (3) SGs 1 g/L, and (4) CCl_4_ 0.5 mL/kg + SGs 1 g/L at 96 h. The following biomarkers were analyzed: lipoperoxidation (LPX), hydroperoxide content (HPC), and protein carbonyl content (PCC), as well as antioxidant activity of superoxide dismutase (SOD) and catalase (CAT). It was found that both (3 and 4) systems' exposure decreases LPX, CHP, PCC, SOD, and CAT with respect to the CCl_4_ system. The results of this study demonstrate that the concentrations of SGs used are not capable of generating oxidative stress and, on the contrary, would appear to induce an antioxidant effect.

## 1. Introduction

High-potency sweeteners are used to provide sweetness of taste without the calories associated with the consumption of sugar [1]. Growing consumption of sugar substitutes has gained importance due to their low caloric intake, potential health benefits, and reduced costs [2]. The most worldwide consumed sweeteners are aspartame (ASP), sucralose (SUC), acesulfame (ACS), saccharin (SAC), cyclamate (CYC), neotame (NEO), alitame (ALI), and neohesperidin dihydrochalcone (NHDC) [3].

Due to the growing incidence of both obesity and type 2 diabetes [4] and health-related concerns such as weight gain [5, 6], cancer risk [7], metabolic syndrome, hypertension, and pregnancy preterminal delivery risks [8–11] associated with the consumption of artificial high-potency sweeteners [12], much attention has been paid to low-calorie plant-derived [13] sucrose substitutes. Nowadays, the food industry is increasingly interested in using natural sugars such as stevia instead of artificial sugars in order to offer a wider range of options for people who do not want or cannot eat sucrose [14].

*Stevia rebaudiana* Bertoni, an herb plant native to Paraguay and Brazil, produces sweet-tasting diterpene compounds as secondary metabolites in its leaves [15]. Stevia is the generic term used for all the compounds or substances derived from the plant* Stevia rebaudiana* Bertoni; nevertheless, the most accurate term for the group of intense sweetening compounds extracted from the plant is steviol glycosides (SGs) [16]. The main SGs found in stevia leaves are stevioside and rebaudioside. These compounds are 250–350 times sweeter than sugar and are widely used in food, beverages, and dietary noncaloric labeled products [17]. As SGs were recognized by the United States' Food and Drug Administration and the European Food Safety Authority as a GRAS (generally recognized as safe) product, it is expected that its global consumption rises to millions of metric tons in the coming years [17].

Globally, the stevia sweetener market is expected to grow from US$ 347 million in 2014 to US$ 565 million by 2020; in terms of volume consumption, stevia is expected to reach almost 8507 tons by the end of 2020 including suppliers such as Nestlé S.A., The Coca-Cola Company, and PepsiCo Inc. and producers such as Cargill Inc., Evolva Holding S.A., Pure Circle Ltd., Stevia Corp., Ingredion Inc., GLG Life Tech Corp., and Tate & Lyle Plc. [18].

Several studies have shown that SGs regulate the content of sugar, radionuclides, and cholesterol in the blood [19]. They also exhibit anti-inflammatory and antitumor promoting properties [20] and insulinotropic, antihyperglycemic, antihypertensive [21–23], and antimicrobial activity [24].

On the other hand, oxidative damage to biological material is inflicted on all compounds of all major chemical classes: proteins, nucleic acids, carbohydrates, and lipids [25]. The inner balance between substances with prooxidant potential and the antioxidant defenses in biological systems [26] can be helpful to assess damage induced by the presence of pollutants in the environment. Carbon tetrachloride (CCl_4_), a well-known hepatotoxic industrial solvent, has been found to provoke damage not only in the liver but also in other tissues such as blood, kidneys, brain, heart, testis, and lungs by generating free radicals [27–29]. Extensive evidence demonstrates that cytochrome P450 metabolic activation of CCl_4_ into free radicals (^*∗*^CCl_4_ and ^*∗*^Cl) induces lipid peroxidation and protein oxidation resulting in severe cell damage [30].

Oxidative stress, which is considered as one of the major mechanisms of action of toxicants, is among the most frequently used biomarkers since it is able to evaluate general damage to biomolecules such as lipids, proteins, and DNA [31]. Oxidative damage to lipids, proteins, and DNA and adverse effects on enzymatic antioxidant defense mechanisms in aerobic organisms have been used in recent years as biomarkers for monitoring environmental pollution [26]. The most important oxidative stress biomarkers used in toxicological studies of aquatic systems are LPX, hydroperoxide content, protein oxidation, and enzymatic antioxidant defenses [32].

Previous studies demonstrate that natural compounds with antioxidant properties may act against oxidative stress induced by CCl_4_ in fish models [33–35]. Therefore, the present study aimed to evaluate the antioxidant activity of SGs on diverse tissues in the common carp* Cyprinus carpio* using oxidative stress (OS) biomarkers. OS will be induced using the CCl_4_ model.

### 1.1. Test Substances

Glycosides of steviol were used: stevioside (13-[(2-O-*β*-D-glucopyranosyl-*β*-D-glucopyranosyl)oxy]kaur-16-en-18-oic acid, *β*-D-glucopyranosyl ester; condensed formula: C_38_H_60_O_18_; CAS number: 57817-89-7; purity of this glycoside was >99%) and rebaudioside-A (13-[(2-O-*β*-D-glucopyranosyl-3-O-*β*-D-glucopyranosyl-*β*-D-glucopyranosyl)oxy]kaur-16-en-18-oic acid, *β*-D-glucopyranosyl ester; condensed formula: C_44_H_70_O_23_; CAS number: 58543-16-1; purity of this glycoside was >97%). These products were provided by Sensient Flavors Mexico.

### 1.2. Fish Procurement and Maintenance

*Cyprinus carpio* species were obtained from a certified aquaculture facility located in Tiacaque, State of Mexico. The carps used for this experiment fit the following characteristics: 15.3 ± 0.58 cm length and 30.01 ± 4.5 g weight. Prior to toxicity studies, organisms were maintained for 30 days in tap water, at 20 ± 2°C, and exposed to natural light/dark photoperiods. Oxygen concentration was kept above 85%, pH at 7.6–7.9, total alkalinity at 17.8 ± 4.3 mg/L, and total hardness at 18.5 ± 0.4 mg/L.

### 1.3. Oxidative Stress Determination

Test systems were prepared using water with the same characteristics and conditions described above in the Fish Procurement and Maintenance. The systems were static without renewal of the medium, and no food was provided to the specimens.

Previous studies were performed by determining the CCl_4_ concentration that induced OS used in this study (0.15, 0.3, 0.44, and 0.62 mL/kg bw) according to Jia et al. [35]. The selected concentration of CCl_4_ was 0.5 mL/kg bw.

In order to determine the concentrations of SGs used in this study, a previous experiment was performed using CCl_4_ 0.5 mL/kg and different concentrations of SGs (70% stevioside and 30% rebaudioside-A proportion): 0.1, 0.2, 0.3, 0.4, 0.5, 0.6, 0.7, 0.8, 0.9, and 1 g/L. And the oxidative stress biomarkers analyzed in this work were evaluated. The only concentration that showed effects on the biomarkers used was 1 g/L of SGs, so this was the one selected.

To test the antioxidant activity of SGs, four systems were tested: (1) SGs free control, (2) CCl_4_ 0.5 mL/kg, (3) SGs 1 g/L, and (4) CCl_4_ 0.5 mL/kg + SGs 1 g/L. Each system used 6 carps and the assays were performed in triplicate (72 fish were used in the oxidative stress evaluation). In the systems containing CCl_4_, the fish were given a caudal vein injection of CCl_4_ (30% in olive oil) at a dose of 0.5 mL/kg body weight. The target concentrations used in this experiment were based on previous experiments (0.29, 0.58, 0.87, and 1.0 g/L SGs). These concentrations were determined by experimental design central composite (STATGRAPHICS Centurion XVII version). The concentration of SGs selected was 1.0 g/L. At the end of the exposure period (96 h), blood was removed by puncture of the caudal vessel, and liver, muscle, gills, and brain were removed from each specimen. Organs and tissues were placed in phosphate buffer solution (PBS) (0.138 M NaCl, 0.0027 KCl (Vetec, Sigma-Aldrich, Mexico)) at pH 7.4 and then centrifuged at 12,500 ×g and −4°C for 15 min. The following biomarkers were then evaluated: hydroperoxide content (HPC), lipoperoxidation (LPX), and protein carbonyl content (PCC), as well as the activity of the antioxidant enzymes superoxide dismutase (SOD) and catalase (CAT). All bioassays were performed on the supernatant.

### 1.4. Determination of HPC

HPC was determined by Jiang et al.'s [36] method. 100 *μ*L of the supernatant (previously deproteinized with 10% trichloroacetic acid; Sigma-Aldrich, St. Louis) was mixed with 900 *μ*L of the reaction mixture [0.25 mM FeSO_4_ (Sigma-Aldrich, St. Louis), 25 mM H_2_SO_4_ (Sigma-Aldrich, St. Louis), 0.1 mM xylenol orange (Sigma-Aldrich, St. Louis), and 4 mM butyl hydroxytoluene (Sigma-Aldrich, St. Louis) in 90% (v/v) methanol (Sigma-Aldrich, St. Louis)]. The mixture was incubated at room temperature for 60 min and absorbance was read at 560 nm against a blank containing only reaction mixture. Results were interpolated on a type curve and expressed as nM CHP (cumene hydroperoxide; Sigma-Aldrich, St. Louis)/mg protein.

### 1.5. Determination of LPX

LPX was determined using thiobarbituric acid-reactive substances, as described by Buege and Aust's [37] method. To 100 *μ*L of supernatant, Tris-HCl buffer solution (pH 7.4) (Sigma-Aldrich, St. Louis) was added until a 1 mL volume was attained. Samples were incubated at 37°C for 30 min; 2 mL of TBA-TCA reagent [0.375% thiobarbituric acid (Fluka-Sigma-Aldrich, Toluca, Mexico) in 15% trichloroacetic acid (Sigma-Aldrich, St. Louis)] was added and samples were shaken. They were then heated up to boiling for 45 min and then allowed to cool down, and the precipitate was removed by centrifugation at 3,000 ×g for 10 min. Absorbance was read at 535 nm against a reaction blank. Malondialdehyde (MDA) content was calculated using the molar extinction coefficient (MEC) of it (1.56 × 10^5^ M/cm). Results were expressed as mM MDA/mg protein.

### 1.6. Determination of PCC

The method of Levine et al. [38] modified by Parvez and Raisuddin [39] and Burcham [40] was used for determining PCC. Soluble proteins were obtained by centrifugation of samples at 10,500 ×g for 30 min. A test tube was filled with 100 *μ*L of supernatant and 150 *μ*L of 10 mM DNPH in 2 M HCl; the tube was incubated at room temperature for 1 h in the darkness. After the incubation time, 500 *μ*L of 20% trichloroacetic acid was added, and the solution was allowed to rest for 15 min at 4°C. The sample was centrifuged for 5 min at 11,000 ×g. Using the solution of 1 : 1 ethanol : ethyl acetate, the bud was washed three times and then dissolved in 1 mL of 6 M guanidine solution (pH 2.3) and incubated for 30 min at 37°C. Absorbance was read at 366 nm. Results were expressed as nM reactive carbonyls formed (C=O)/mg protein, using the MEC of 21,000 M/cm [41].

### 1.7. SOD Activity Determination

According to the Misra and Fridovich's method [42], the activity of SOD was determined. In a 1 cm cuvette, 40 *μ*L of the supernatant, 200 *μ*L of adrenaline (30 mM), and 260 *μ*L of carbonate buffer solution (50 mM sodium carbonate and 0.1 mM EDTA) (pH 10.2) were added. Absorbance was read at 480 nm after 30 s and 5 min. SOD activity was determined using the MEC of SOD (21 M/cm). Results were expressed as IU SOD/mg protein [41].

### 1.8. CAT Activity Determination

According to Radi et al.'s [43] method, the activity of CAT was determined. A test tube was filled with 20 mL of the supernatant, followed later by addition of 1 mL of isolation buffer solution [0.3 M saccharose (Vetec-Sigma-Aldrich, St. Louis), 1 mL EDTA (Sigma-Aldrich, St. Louis), 5 mM HEPES (Sigma-Aldrich, St. Louis), and 5 mM KH_2_PO_4_ (Vetec-Sigma-Aldrich, St. Louis)], plus 0.2 mL of a hydrogen peroxide solution (20 mM, Vetec-Sigma-Aldrich, St. Louis). Absorbance was read at 240 nm after 0 and 60 s. The absorbance value was used in the formula CAT concentration = (*A*_0_ − *A*_60_)/MEC, where the MEC of H_2_O_2_ is 0.043 mM/cm, and the results were expressed as *μ*M H_2_O_2_/mg protein [41].

### 1.9. Determination of Total Protein

25 *μ*L of the supernatant was mixed with 75 *μ*L of deionized water and 2.5 mL of Bradford's reagent. The mix was shaken in a vortex for 1 min and then stored without light for 5 min. Absorbance was read at 595 nm and the results were interpolated on a bovine albumin curve. Total protein analysis was determined by the Bradford [44] method.

### 1.10. Statistical Analysis

Results of the oxidative stress biomarkers were statistically evaluated by one-way analysis of variance (ANOVA), followed by Bonferroni's multiple comparisons test, with *P* set at <0.05. Statistical determinations were performed with SPSS v10 software (SPSS, Chicago, IL, USA).

## 2. Results

### 2.1. Hydroperoxide Content (HPC)

**Figure 1:**
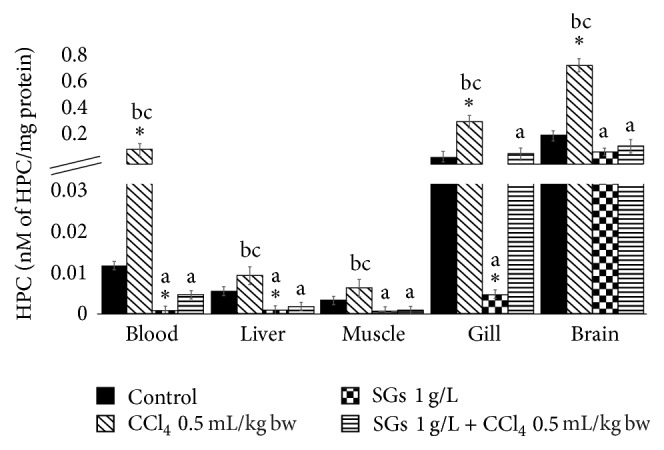
HPC in blood, liver, muscle, gill, and brain of* C. carpio* exposed at 96 h in different systems. Values are the mean of three replicates ± SEM. *N* = 72 carps. CHP: cumene hydroperoxide. Significantly different (*P* < 0.05) from *∗* = control, a = CCl_4_, b = SGs, and c = SGs + CCl_4_. ANOVA and Bonferroni's test.

The amount of cumene hydroperoxide (CHP) equivalents induced by different systems is shown in Figure 1. Significant increases with respect to the control (*P* < 0.05) were observed in the treatment with CCl_4_ in blood, gill, and brain in 892.1, 457.1, and 250.4%, respectively. In the SGs system, significant decreases were observed with respect to the control (*P* < 0.05) in blood (91.3%), liver (82.9%), and gill (94.4%). No significant differences were observed in the treatment with SGs + CCl_4_ in any tissue. Significant decreases (*P* < 0.05) with respect to the CCl_4_ were found in all tissues in the SGs system. The SGs + CCl_4_ system showed significant decreases (*P* < 0.05) with respect to the CCl_4_ system in blood, liver, muscle, gill, and brain in 96.0, 80.9, 83.7, 76.2, and 81.7%, respectively.

### 2.2. Lipoperoxidation (LPX)

**Figure 2:**
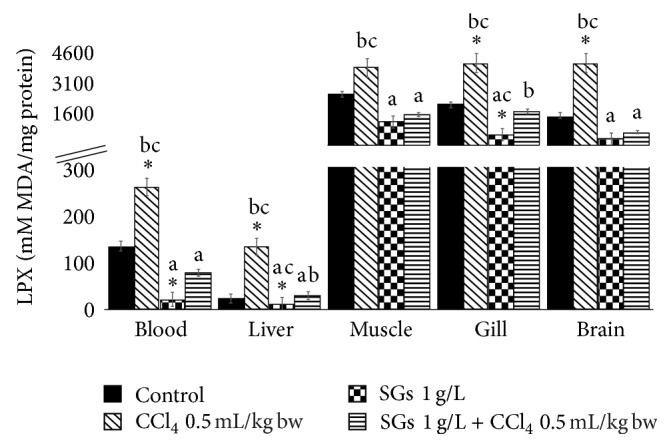
LPX in blood, liver, muscle, gill, and brain of* C. carpio* exposed at 96 h in different systems. Values are the mean of three replicates ± SEM. *N* = 72 carps. MDA: malondialdehyde. Significantly different (*P* < 0.05) from *∗* = control, a = CCl_4_, b = SGs, and c = SGs + CCl_4_. ANOVA and Bonferroni's test.

LPX results are shown in Figure 2. Significant increases with respect to the control group (*P* < 0.05) were observed in the system with CCl_4_ in blood, liver, gill, and brain. These increases were 92.6, 434.9, 99.9, and 171.5%, respectively. Significant decreases with respect to the control group (*P* < 0.05) were observed in the system with SGs in blood, liver, and gill. The decreases observed were 83.7, 91.1, and 72.3%, respectively. No significant differences were observed in the treatment with SGs + CCl_4_ in any tissue. Significant decreases with respect to the CCl_4_ system were found in blood (91.5%), liver (91.0%), muscle (68.9%), gill (86.1%) and brain (90.6%) in the SGs system. Significant decreases with respect to the CCl_4_-induced system were found in blood, liver, muscle, gill, and brain in the SGs + CCl_4_ system. The decreases observed were 69.7, 76.9, 60.0, 58.7, and 83.2%, respectively.

### 2.3. Protein Carbonyl Content (PCC)

**Figure 3:**
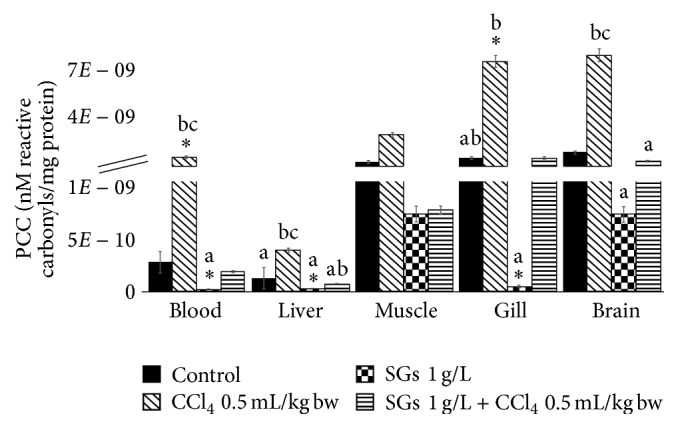
PCC in blood, liver, muscle, gill, and brain of* C. carpio* exposed at 96 h in different systems. Values are the mean of three replicates ± SEM. *N* = 72 carps. Significantly different (*P* < 0.05) from *∗* = control, a = CCl_4_, b = SGs, and c = SGs + CCl_4_. ANOVA and Bonferroni's test.

PCC results are shown in Figure 3. Significant increases with respect to the control group (*P* < 0.05) were observed in the CCl_4_ system in blood (514.6%) and gill (417.9%). Exposition to SGs (1 g/L) showed significant decreases (*P* < 0.05) with respect to the control in blood, liver, and gill. These decreases were 91.9, 75.6, and 97.0%, respectively. No significant differences were observed in the treatment with SGs + CCl_4_ in any tissue. No significant differences of the biomarker were observed in muscle in any treatment. Significant decreases with respect to the CCl_4_-induced system were observed in the SGs system in blood, liver, gill, and brain. Significant differences were observed in the SGs + CCl_4_ system with respect to the CCl_4_ system in blood (88.9%), liver (80.2%), gill (80.4%), and brain (84.0%). No significant differences were observed in muscle in any treatment.

### 2.4. Superoxide Dismutase (SOD) Activity

**Figure 4:**
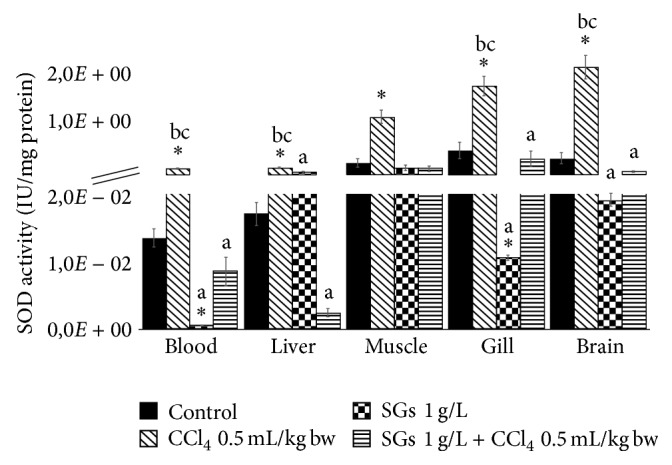
SOD activity in blood, liver, muscle, gill, and brain of* C. carpio* exposed at 96 h in different systems. Values are the mean of three replicates ± SEM. *N* = 72 carps. Significantly different (*P* < 0.05) from *∗* = control, a = CCl_4_, b = SGs, and c = SGs + CCl_4_. ANOVA and Bonferroni's test.

SOD activity is shown in Figure 4. CCl_4_ treatment showed significant increases in all tissues (blood, liver, muscle, gill, and brain) with respect to control (*P* < 0.05). These increases were 951.9, 672.6, 401.2, 272.3, and 562.6%, respectively. SGs exposition showed significant decreases (*P* < 0.05) with respect to control in blood (96.1%) and gills (97.8%). A significant increase was observed in the liver (166.3%) with the SGs treatment with respect to the control group. No significant differences were observed in the treatment with SGs + CCl_4_ in any tissue. Significant decreases with respect to the CCl_4_ system were observed in blood, liver, and gill in the SGs system. Decreases observed were 99.6, 65.5, and 99.4%, respectively. Significant decreases in the SGs + CCl_4_ system were observed in blood (94.0%), liver (98.2%), gill (82.3%), and brain (96.9%) with respect to the CCl_4_ system.

### 2.5. Catalase (CAT) Activity

**Figure 5:**
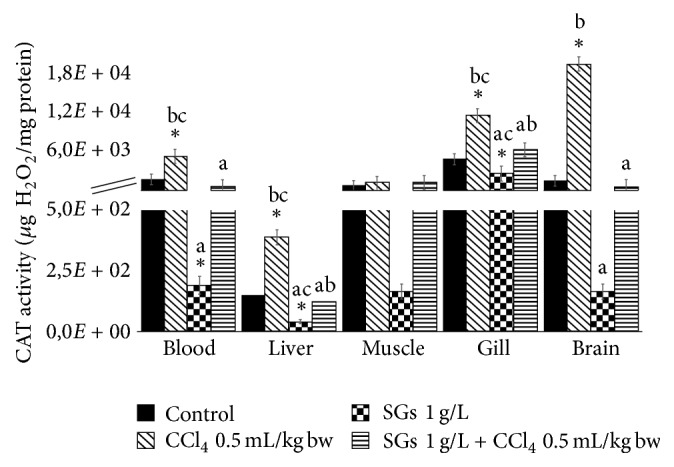
CAT enzymatic activity in blood, liver, muscle, gill, and brain of* C. carpio* exposed at 96 h in different systems. Values are the mean of three replicates ± SEM. *N* = 72 carps. Significantly different (*P* < 0.05) from *∗* = control, a = CCl_4_, b = SGs, and c = SGs + CCl_4_. ANOVA and Bonferroni's test.

CAT activity is shown in Figure 5. In the CCl_4_ system, significant increases were observed with respect to the control group (*P* < 0.05) in blood, liver, gill, and brain (203.6, 161.9, 139.8, and 1233.8%, resp.). Significant decreases with respect to the control group were found in blood (88.3%), liver (72.6%), and gill (46.2%) in the SGs system. No significant differences of the biomarker were observed in muscle in any treatment. No significant differences were observed in the treatment with SGs + CCl_4_. Significant decreases were found in the SGs system in blood (96.1%), liver (89.5%), gill (77.6%), and brain (99.1%) with respect to the CCl_4_ system. Significant decreases were found in the SGs + CCl_4_ system in blood, liver, gill, and brain with respect to the CCl_4_ system. These decreases were 88.0, 67.8, 45.4, and 97.1%, respectively. Significant increases were observed in liver and gill with respect to the SGs system in the SGs + CCl_4_ system.

## 3. Discussion

*Stevia rebaudiana* Bertoni, a plant species native to Northeastern Paraguay, is known to accumulate diterpene glycosides. These compounds are nontoxic, high-potency sweeteners that are used as sugar substitutes. The major SGs stevioside and rebaudioside-A constitute 60–70% and 20–30%, respectively, of the total glycosides in* S. Rebaudiana*. Several studies have suggested that, besides sweetness, SGs, along with related compounds, may also offer therapeutic benefits, as they have antihyperglycemic, antihypertensive, anti-inflammatory, antitumor, antidiarrheal, diuretic, and immunomodulatory effects [4].

Another important effect of SGs related to the scientific literature is their antioxidant activity. In a study conducted by Shukla et al. [45], they demonstrated that aqueous extracts also inhibited hydroxyl radicals, nitric oxide, and superoxide anions with IC_50_ values of 100.86, 98.73, and 100.86 *μ*g/mL, respectively. The greater amount of phenolic compounds leads to more potent radical scavenging effects as shown by the aqueous leaf extract of* S. rebaudiana*.

OS is one important mechanism of toxicity, given the impact that an imbalance between reactive oxygen species (ROS) and antioxidant defenses has on vital biomolecules such as lipids, proteins, and genetic material, as well as the countless toxicants capable of inducing it (hydrocarbons, metals, pesticides, solvents, and drugs, among other compounds) [46–51]. An increase in the scientific literature suggests that diseases including cardiovascular diseases, diabetes, cancer, neural disorders, arthritis, and aging are caused by or related to the production of ROS which can result in tissue damage and cell death [52–54]. Kim et al. [55] reported that* Stevia* extracts contain high levels of compounds with ROS scavenging activity.

On the other hand, sugars are well known as ROS scavengers [56] and a number of recent findings point to a prominent role for sugars or sugar-like compounds in oxidative stress defense in plants [55–57]. A study conducted by Hajihashemi and Geuns [58] hypothesized that, due to the high content of sugars present in the SGs, these presented their high antioxidant capacity; however, the results showed that this did not occur.

In the same way as all aerobic organisms, fish such as* Cyprinus carpio* have an inherent and efficient antioxidant defense system that includes the enzymes SOD, CAT, and GPx and nonenzymatic antioxidant components such as GSH [59]. These enzymes play a preponderant role in defending the cells against free radical-mediated oxidative damage [60] decreased activities, or expressions of these enzymes may predispose tissues to free radical injury [61].

From the above, this study was carried out to investigate the antioxidant properties of SGs in CCl_4_-induced injuries in a fish model* (Cyprinus carpio)*. The utilization of halogenated alkanes such as CCl_4_, CHCl_3_, or CHI_3_ has been prohibited due to their severe toxicity; CCl_4_ however continues to be used as a model substance to elucidate the mechanisms of action of hepatotoxic effects such as fatty degeneration, fibrosis, hepatocellular death, carcinogenicity, and OS. The mechanism through which CCl_4_ produces its toxic effects is the formation of reactive trichloromethyl radicals (^*∙*^CCl_3_) by CYP450 activity. In the presence of oxygen, ^*∙*^CCl_3_ is quickly transformed into trichloromethyl peroxyl radical (CCl_3_O_2_^⁡∙^). CCl_3_O_2_^⁡∙^ binds covalently to cellular proteins or lipids, which initiates the lipid peroxidation in the cellular membrane [62].

In the current work, treatment of the fish with CCl_4_ at 96 h increased significantly the activities of SOD and CAT in all tested tissues. In addition, it increased significantly CHP, LPX, and PCC in blood, liver, gill, and brain. These results demonstrate that CCl_4_ may be a good inducer of OS in common carp* Cyprinus carpio*.

Antioxidant defenses may be induced by diverse environmental contaminants [63]. SOD is the first mechanism of antioxidant defense and the main enzyme responsible for offsetting the toxic effects induced by the presence of ROS. This is particularly so in the case of the superoxide ion, which is a minor product of mitochondrial respiration [64] and is biotransformed by SOD to hydrogen peroxide, after which CAT and GPx take part in the capture and later dismutation of H_2_O_2_ to H_2_O [31]. As can be seen in Figures 4 and 5, CCl_4_ at a concentration of 0.5 mL/kg bw increases significantly the SOD and CAT activity in blood and the four tissues evaluated in this study. Also, there are increases in HPC, LPX, and PCC. Bagnyukova et al. [65] say that LPX products are apparently involved in the upregulation of certain antioxidant enzymes. Therefore, LPX increases in our study might likewise explain the increases in antioxidant enzyme activities that were found. These results would demonstrate that CCl_4_ is a good inducer of oxidative stress in common carp.

SGs alone system showed an efficient decrease in the oxidative stress biomarkers used in this study with respect to control and CCl_4_ systems; and to determine the effect of CCl_4_ exposure on OS system and consequently potential antioxidant effects of SGs on the disturbed system by CCl_4_, a system was tested using CCl_4_ 0.5 mL/kg + SGs 1 g/L. The results in this system showed a significant decrease in the cellular oxidation biomarkers (CHP, LPX, and PCC) and the antioxidant enzymes (SOD and CAT) with respect to the CCl_4_ systems.

These results are in agreement with those of Holvoet et al. [66]; they demonstrate that stevioside treatment of obese diabetic mice improved adipose tissue maturation and increased glucose transport, insulin signaling, and antioxidant defense in white visceral adipose tissues. Together, these increases were associated with a twofold increase of adiponectin. The adiponectin has been associated with improved insulin signaling and antioxidant defense in both the adipose tissue and the aorta of stevioside-treated mice [67].

Also, these authors showed that rebaudioside increased methionine that is directly involved in the regulation of the glutathione antioxidative system. It also increased tryptophan that is involved in the regulation of the defense system through its action as a precursor of antioxidants and its effect on the inflammatory response [68].

In addition to these findings, it was shown that SGs had a very potent ^*∙*^OH scavenging activity [69, 70]. Several studies have shown that crude extracts of* Stevia rebaudiana* are responsible for antioxidant activities in murine models, neutralizing radicals such as hydroxyl radicals (OH^*∗*^), superoxide radicals (O^2*∗*^), and hydrogen peroxide (H_2_O_2_).

On the other hand, when SGs were used, a decrease in HPC, LPX, and PCC (Figures 1–3) was observed with respect to the control group and the CCl_4_ group at concentration of 0.5 mL/kg bw. Paradoxically, the levels of antioxidant enzymes SOD and CAT decreased significantly with respect to the control, CCl_4_ at concentration of 0.5 mL/kg bw, and SGs + CCl_4_ groups. The decrease in SOD and CAT of the CCl_4_ and SGs + CCl_4_ groups would demonstrate that the increase of ROS by tetrachloride exposure in common carp is inhibiting the antioxidant enzymes evaluated. Jira et al. [71] suggest that SOD activity may be inhibited if there is an increase in superoxide anion, H_2_O_2_, and peroxynitrite.

In the group in which only SGs were used, a significant decrease with respect to the control was observed, which would demonstrate that the glycosides of steviol are effective in reducing the ROS produced by the basal cellular activity of the carp.

## 4. Conclusion

SGs used alone are not capable of generating oxidative stress and, on the contrary, would appear to induce an antioxidant effect in the common carp* Cyprinus carpio*. The antioxidant properties of SGs in CCl_4_-induced injuries in* Cyprinus carpio* model were proven when comparing the systems with CCl_4_ alone and the mixture of CCl_4_ and SGs system. The concentration of SGs which showed an antioxidant activity in the model used was 1 g/L.
